# Random Transfer of *Ogataea polymorpha* Genes into *Saccharomyces cerevisiae* Reveals a Complex Background of Heat Tolerance

**DOI:** 10.3390/jof7040302

**Published:** 2021-04-15

**Authors:** Taisuke Seike, Yuki Narazaki, Yoshinobu Kaneko, Hiroshi Shimizu, Fumio Matsuda

**Affiliations:** 1 Department of Bioinformatics Engineering, Graduate School of Information Science and Technology, Osaka University, 1-5 Yamadaoka, Suita, Osaka 565-0871, Japan; taisuke_seike@ist.osaka-u.ac.jp (T.S.); navynarazaki0911@gmail.com (Y.N.); shimizu@ist.osaka-u.ac.jp (H.S.); 2 Yeast Genetic Resources Laboratory, Graduate School of Engineering, Osaka University, Osaka 565-0871, Japan; kaneko@bio.eng.osaka-u.ac.jp

**Keywords:** *Saccharomyces cerevisiae*, cDNA library, heat tolerance, random gene transfer, *Ogataea polymorpha*

## Abstract

Horizontal gene transfer, a process through which an organism acquires genes from other organisms, is a rare evolutionary event in yeasts. Artificial random gene transfer can emerge as a valuable tool in yeast bioengineering to investigate the background of complex phenotypes, such as heat tolerance. In this study, a cDNA library was constructed from the mRNA of a methylotrophic yeast, *Ogataea polymorpha*, and then introduced into *Saccharomyces cerevisiae*. *Ogataea polymorpha* was selected because it is one of the most heat-tolerant species among yeasts. Screening of *S. cerevisiae* populations expressing *O. polymorpha* genes at high temperatures identified 59 *O. polymorpha* genes that contribute to heat tolerance. Gene enrichment analysis indicated that certain *S. cerevisiae* functions, including protein synthesis, were highly temperature-sensitive. Additionally, the results confirmed that heat tolerance in yeast is a complex phenotype dependent on multiple quantitative loci. Random gene transfer would be a useful tool for future bioengineering studies on yeasts.

## 1. Introduction

Horizontal gene transfer is a rare evolutionary event in yeast, in which functional genes are acquired from other species [1]. Gene transfer among eukaryotes is a relatively rare event that is limited by unknown barriers [2,3,4]. Genome analysis revealed that the budding yeast *Saccharomyces cerevisiae* has acquired several genes from bacteria, such as those encoding metabolite enzymes and transporters [5,6,7].

Artificial random gene transfer is a technique used in conventional genetic engineering to identify valuable genes, such as those useful for metabolic engineering [8]. In addition, the technique can help investigate the genetic background of quantitative traits, as many loci, each with small effects, contribute to heat tolerance [9,10]. Transfer of a gene pool would provide a list of genes that contribute to heat tolerance in the host yeast, and this list of genes will help elucidate the genetic complexity behind the heat tolerance phenotype.

In this study, we used a cDNA library for a random gene transfer experiment [11]. The cDNA library was constructed from the mRNA of a methylotrophic yeast, *Ogataea polymorpha*, and then introduced into *S. cerevisiae*. *Saccharomyces cerevisiae* is an industrial host for bioethanol production. Since improved heat tolerance reduces the costs required for cooling during fermentation [12], many genetic analyses and adaptive evolutionary studies have been performed [13,14,15]. *Ogataea polymorpha* was selected because it can grow at temperatures close to 50 °C and is one of the most heat-tolerant species among yeasts [16,17]. Under high temperatures, screening the *S. cerevisiae* population identified 60 colonies showing improved heat tolerance and the corresponding *O. polymorpha* genes responsible for heat tolerance. Additionally, the list of genes uncovered the *S. cerevisiae* functions that are sensitive to high temperatures.

## 2. Materials and Methods

### 2.1. Strains, Plasmids, and Yeast Transformation

The yeast strains and plasmids used in this study are listed in Table 1. Plasmids were derived from pGK413, pGK414, or pGK416, in which gene expression is controlled by the PGK1 promoter [18]. For the construction of cDNA libraries, *O. polymorpha* BY4329 cells were cultured till the exponential growth phase in 5 mL of yeast extract-peptone-adenine-dextrose (YPAD) medium and then harvested by centrifugation at 12,000× *g* for 5 min. mRNA was extracted from the cells using the Ribo-Pure Yeast Kit (Thermo Fisher Scientific, Waltham, MA, USA). Two overlapping regions for the In-Fusion method were added to the three plasmids using the inverse PCR method with pGK413, pGK414, and pGK416 as the templates and the primers pGK_inv_fw (TCTCATCGTACCCCGGAAATAAATT) and pGK_inv_rv (AACTATGGTGACGAAGTTTTATATTTGTTG) [11]. A cDNA library was constructed from the mRNA mixture and the amplicon of inverse PCR, using the In-Fusion SMARTer Directional cDNA Library Construction Kit (Takara Bio, Inc., Shiga, Japan). The In-Fusion mixture was introduced into *Escherichia coli* HST08 competent cells (Takara Bio, Inc.) by electroporation at 25 μF and 2 kV. The pulse controller was set to 200 Ω using a GenePulser (Bio-Rad Laboratories, Hercules, CA, USA). Ampicillin-resistant cells were recovered from agar plates, from which the plasmid pools were prepared. The growth conditions, DNA-related techniques, and the lithium-acetate method for transformation have been described previously [19].

### 2.2. Culture Conditions

All strains were cultured in YPAD medium (1% Bacto yeast extract, 2% Bacto peptone, 2% glucose, and 0.004% adenine) and synthetic dextrose (SD) medium (0.67% yeast nitrogen base without amino acids and 2% or 0.5% glucose, as necessary, 0.006% leucine, 0.003% lysine hydrochloride, 0.002% histidine, 0.004% adenine, 0.004% tryptophan, and 0.002% uracil). Yeast cells grown on the agar plate were cultured in 5 mL of SD medium containing the required amino acids overnight at 30 °C and 150 rpm. To screen for cell growth, the transformants were cultured overnight in SD agar medium containing amino acids at 39 °C or 39.5 °C in an incubator (TVA360DB, ADVANTEC, Tokyo, Japan). 

### 2.3. Construction of Screening System for the Heat-Resistant Evolved Strain

*Saccharomyces cerevisiae* YPH499 was transformed with the cDNA library of *O. polymorpha* using the lithium-acetate method and then cultured for several days in SD agar medium to obtain colonies of transformants. From the original SD agar plates, replica plates were prepared on other SD agar plates using the replica plating method. The replicas were cultured at 39 °C or 39.5 °C. The plasmids in the selected transformants were extracted using the Easy Yeast Plasmid Isolation Kit (Takara). Each plasmid was introduced into *E. coli* HST08 competent cells and cultured in L medium containing 5 mL ampicillin. Plasmid purification was performed using LaboPass Mini (Hokkaido System Science), and sequence analysis was performed using the PGK 5′ primer (TAGTTTTTCAAGTTCTTAGA) and PGK 3′ primer (CTATTATTTTAGCGTAAAGG). For each plasmid, the corresponding *O. polymorpha* gene was identified using the BLAST search function in the UniProt database, including *O. polymorpha* genome information (http://www.uniprot.org/ accessed on 13 April 2021) [17]. *Saccharomyces cerevisiae* orthologs were identified using the BLAST search of the Saccharomyces Genome Database (SGD, https://www.yeastgenome.org/ accessed on 13 April 2021). Gene enrichment analysis was performed using the over-representation analysis function of the WebGestalt web tool (http://www.webgestalt.org/ accessed on 13 April 2021) [20]. The Gene Ontology (GO) dataset of all *O. polymorpha* proteins was retrieved from the UniProt database. The Benjamini-Hochberg (GH) method was used to evaluate the false discovery rate (FDR).

### 2.4. Confirmation of Reproducibility by Spot Method

Transformants were inoculated on SD agar medium containing 20 g/L glucose and cultured at 30 °C for two days. A single colony grown on the plate was inoculated into a test tube containing 5 mL of SD medium and precultured at 30 °C and 150 rpm. The preculture solution was then centrifuged at 3000 rpm and 4 °C. The collected transformants were suspended in sterile distilled water. Suspensions (6 μL) were then spotted onto SD agar medium supplemented with the appropriate amino acids and incubated at 39 °C or higher for five days. 

## 3. Results

### 3.1. Comparison of Vectors for Artificial Random Gene Transfer 

**Figure 1:**
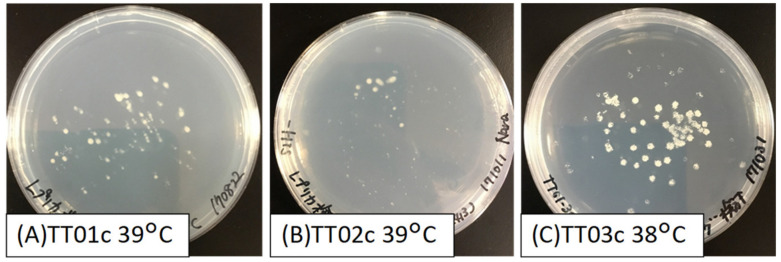
Colonies on replica plates of control strains cultivated under high temperature conditions for one week. (**A**) TT01c (YPH499 (pGK416)) cultured at 39 °C. (**B**) TT02c (YPH499 (pGK413)) cultured at 39 °C. (**C**) TT03c (YPH499 (pGK414)) cultured at 38 °C.

For the construction of an *O. polymorpha* cDNA library, three CEN/ARS plasmid vectors (single copy-type), namely pGK416 (possessing *URA3*), pGK413 (possessing *HIS3*), and pGK414 (possessing *TRP1*), were employed [18]. Because the relationship between amino acid auxotrophy and heat tolerance was expected, three control strains possessing pGK416, pGK413, and pGK414 (strains TT01c, TT02c, and TT03c, respectively) were constructed from the *S. cerevisiae* YPH499 strain and cultured on agar plates to compare their heat tolerance phenotypes (Figure 1A,B). The TT01c and TT02c strains were able to grow at 39 °C but failed to grow at 39.5 °C. However, many colonies that grew at 39 °C showed an abnormally wet phenotype. The upper growth limit of TT03c was 38 °C (Figure 1C). These results showed that the amino acid auxotrophy of *S. cerevisiae* affected the heat tolerance of yeast for as yet unknown reasons.

### 3.2. Screening of Heat-Tolerant S. cerevisiae Strains Expressing O. polymorpha cDNA

A cDNA library was constructed from the *O. polymorpha* BY4329 strain. The cDNA fragments were inserted into pGK416, pGK413, and pGK414, which were then introduced into the *S. cerevisiae* YPH499 strain to produce three populations possessing *O. polymorpha* cDNA (TT01, TT02, and TT03, respectively). Approximately 400 colonies grew on each selection plate, and a replica plate was prepared using the replica plating technique. A total of 50–80 replica plates consisting of approximately 20,000–32,000 colonies were prepared for each population (TT01, TT02, and TT03).

The replica plates were incubated at 39.5 °C for the TT01 and TT02 populations and at 39 °C for the TT03 population. After one week, no colonies were obtained from the TT02 population. In contrast, 11 and 49 colonies were obtained on the replica plates of the TT01 and TT03 populations, respectively. No colonies were identified after additional screening at higher temperatures.

Following the collection of plasmid vectors from the 60 colonies, sequences of open reading frames of the cDNAs were determined to identify the corresponding *O. polymorpha* genes, *S. cerevisiae* ortholog genes, and their putative functions using the BLAST search of UniProt and SGD databases (Table 2 and Table S1) [17]. Among the 60 colonies, an identical gene (*OGAPODRAFT_52470*, an ortholog of *S. cerevisiae QCR8* ubiquinol-cytochrome c reductase subunit 8) was identified from two independent colonies (TT01-2 and TT01-8). The cDNAs obtained from four colonies (TT03-46, -47, -48, and -49) had poor homology to all *S. cerevisiae* ORFs (E-value < 1.0 × 10^−3^), suggesting that these cDNAs were derived from *O. polymorpha*-specific genes.

Functional categorization of the annotation list revealed that the transferred cDNAs encoded genes involved in various functions such as metabolism (for example, *HPODL_02693* encoding 6-phosphofructo-2-kinase, obtained from colony ID TT03-21), translation (*HPODL_00942* encoding ribosomal protein P2B, obtained from colony ID TT03-29), electron transport chain (*HPODL_02610* encoding cytochrome c1, obtained from colony ID TT01-9), and protein quality control (*OGAPODRAFT_12972* encoding the chaperonin GroES, obtained from colony ID TT01-7).

Gene enrichment analysis was performed using the GH method to control the FDR. The results showed that genes encoding ribosomal proteins and other proteins involved in translation were overrepresented in the list of 59 *O. polymorpha* genes, with 11 (19%) and 9 (15%) cDNAs encoding genes related to the GO terms “structural constituent of ribosome” and “translation”, respectively (Table 3).

### 3.3. Reconstruction of Heat-Tolerant S. cerevisiae Strains

**Figure 2:**
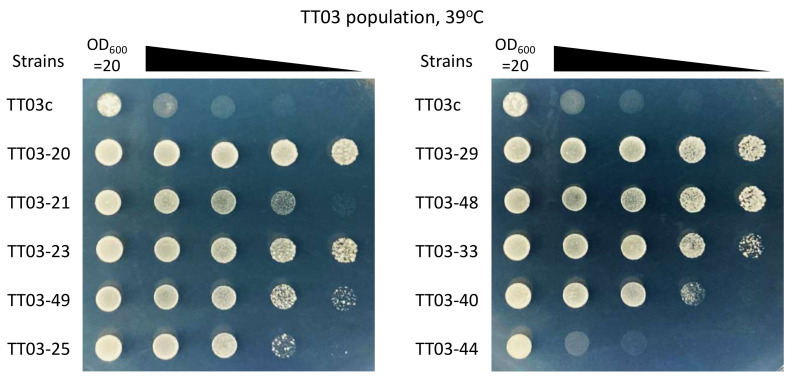
Heat tolerance of reconstructed strains. The dilution series was employed to confirm differences in heat tolerance. Samples were withdrawn from cultures, and their OD_600_ values were adjusted to 20. Five-fold serial dilutions of these cultures were prepared in sterile distilled water, and 6 μL each of the cultures at OD_600_ = 20 and their dilutions were spotted onto SD plates without tryptophan. The plates were then incubated at 39 °C for 5 days and photographed.

The *S. cerevisiae* strains listed in Table 2 were reconstructed to check the false-positive rate derived from the screening approach. For this purpose, 10 out of the 49 plasmid vectors were randomly selected from the TT03 populations and then introduced into the YPH499 strains. The heat tolerance of the reconstructed strains was investigated using a spot assay under high temperature conditions (Figure 2). Although 5-fold serial dilutions were employed to confirm differences in heat tolerance, we found that almost all reconstructed strains tended to be more tolerant to high temperatures (39 °C) than the control strain (TT03c). These results suggest that the false-positive rate was low enough in the screening approach.

## 4. Discussion

In this study, we introduced a cDNA library derived from *O. polymorpha* into *S. cerevisiae.* Screening of the *S. cerevisiae* populations expressing the *O. polymorpha* cDNA library under high temperature conditions resulted in 60 colonies showing improved heat tolerance and identification of the *O. polymorpha* genes responsible for heat tolerance (Figure 2 and Table 2). These results reveal three aspects of the high temperature tolerance of *S. cerevisiae*.

First, we identified 59 candidate genes in *O. polymorpha* that contribute to heat tolerance. However, these results do not imply that the heat stability of proteins is derived from these genes. Since a strong promoter (PGK1 promoter) was used to express cDNA, a large amount of overexpressed proteins might have also contributed to heat tolerance. Further genetic and biochemical characterization is needed to examine the heat stability of the proteins expressed from the candidate genes. Moreover, this experiment failed to transfer all *O. polymorpha* genes to *S. cerevisiae* because the cDNA library used in this study was collected from *O. polymorpha* in exponential growth phase at 30 °C. More candidate genes are likely to be obtained using more comprehensive cDNA libraries prepared from *O. polymorpha*, for instance, under high temperature conditions.

Second, the genes identified in this study revealed the *S. cerevisiae* functions that are sensitive to high temperatures, because these functions were complemented by the expression of corresponding genes derived from *O. polymorpha.* Gene enrichment analysis showed that many of the *S. cerevisiae* proteins sensitive to high temperatures were ribosomal proteins and those involved in other steps of translation. Previous studies have reported that genes related to chaperonins [21], superoxide dismutase [22], ubiquitination [23], nitric oxide [24], H^+^-ATPase [25], and trehalose biosynthesis [26,27] were responsible for the heat tolerance of *S. cerevisiae*. While genes encoding chaperonin (TT01-7) and superoxide dismutase (TT03-20) were found, genes responsible for other functions such as H^+^-ATPase activity and trehalose biosynthesis were not found in the present study (Table 2). These results indicate that chaperonins, superoxide dismutase, ribosome, and translation may be additional targets for improving the heat tolerance of *S. cerevisiae*.

Thirdly, our results highlight that heat tolerance in yeasts is a complex phenotype that is controlled by multiple genes. This supports the idea that the improvement of heat tolerance in *S. cerevisiae* requires the expression of multiple heat-stable proteins. This study demonstrated that random gene transfer is a helpful laboratory evolution tool for investigating the genetic background of complex phenotypes, as well as for enabling future bioengineering studies.

## Figures and Tables

**Table 1:** Strains and plasmids used in this study.

Strain Name	Genotype	Source
*Ogataea polymorpha* BY4329	*Leu1-1*	Obtained from NBRP Yeast
*Saccharomyces cerevisiae* YPH499	*MATa, ura3-52 lys2-801_amber ade2-101_ochre trp1-Δ63 his3-Δ200 leu2-Δ1*	Thermo Scientific
TT01	YPH499 (pGK416_BY4329 cDNA library)	This study
TT02	YPH499 (pGK413_BY4329 cDNA library)	This study
TT03	YPH499 (pGK414_BY4329 cDNA library)	This study
TT01c	YPH499 (pGK416)	This study
TT02c	YPH499 (pGK413)	This study
TT03c	YPH499 (pGK414)	This study
*Escherichiacoli* DH5α	*deoR endA1 gyrA96 hsdR17*(*rk-mk+) recA1 relA1 supE44 thi-1Δ*(*lacZYA-argFV169*) φ80*lacZΔM15* F-	
*Escherichiacoli* HST08	*F,endA1, supE44, thi-1, recA1, relA1, gyrA96, phoA, Φ80d lacZΔM15, Δ(lacZYA-argF) U169, Δ(mrr-hsdRMS-mcrBC), ΔmcrA,λ–*	
Plasmids		
pGK413	Yeast expression vector containing PGK1 promoter, origin, ARS4/CEN6 HIS3 marker, no expression (control plasmid)	[18]
pGK414	Yeast expression vector containing PGK1 promoter, origin, ARS4/CEN6 TRP1 marker, no expression (control plasmid)	[18]
pGK416	Yeast expression vector containing PGK1 promoter, origin, ARS4/CEN6 URA3 marker, no expression (control plasmid)	[18]

**Table 2:** Annotation of *O. polymorpha* genes obtained from colonies of heat-tolerant *S. cerevisiae* expressing *O. polymorpha* cDNA ^(1)^.

Colony ID	Gene ID of *O. polymorpha* ^(2)^	*S. cerevisiae* Ortholog ^(3)^	Functional Annotation of *S. cerevisiae* Ortholog
TT01-1	*OGAPODRAFT_7331*	*CAF20*	cap-associated protein CAF20
TT01-2	*OGAPODRAFT_52470*	*QCR8*	ubiquinol-cytochrome c reductase subunit 8
TT01-3	*OGAPODRAFT_16764*	*ALD4*	aldehyde dehydrogenase
TT01-4	*HPODL_02546*	*RPL16A*	60S ribosomal protein L16-B
TT01-5	*HPODL_00806*	*GUP1*	acyltransferase
TT01-6	*OGAPODRAFT_17522*	*THO1*	SAP domain-containing ribonucleoprotein
TT01-7	*OGAPODRAFT_12972*	*HSP10*	chaperonin GroES
TT01-8	*OGAPODRAFT_52470*	*QCR8*	ubiquinol-cytochrome c reductase subunit 8
TT01-9	*HPODL_02610*	*CYT1*	cytochrome c1, heme protein, mitochondrial
TT01-10	*HPODL_04437*	*FRK1*	serine/threonine protein kinase
TT01-11	*OGAPODRAFT_15309*	*PAF1*	RNA polymerase II-associated factor 1
TT03-1	*HPODL_02637*	*GRS1*	glycine--tRNA ligase 1, mitochondrial
TT03-2	*HPODL_00026*	*NAP1*	histone chaperone NAP1
TT03-3	*HPODL_05027*	*NAB2*	mRNA-binding protein NAB2
TT03-4	*HPODL_03235*	*ERV25*	p24 family protein delta-1
TT03-5	*HPODL_05028*	*RPS2*	ribosomal 40S subunit protein S2
TT03-6	*OGAPODRAFT_25583*	*RIB3*	3,4-dihydroxy-2-butanone-4-phosphate synthase RIB3
TT03-7	*HPODL_03162*	*ACB1*	long-chain fatty acid transporter ACB1
TT03-8	*HPODL_01585*	*RAD4*	DNA repair protein RAD4
TT03-9	*HPODL_00194*	*MRP7*	mitochondrial 54S ribosomal protein YmL2
TT03-10	*HPODL_02367*	*RPS31*	ubiquitin-ribosomal 40S subunit protein S31 fusion protein
TT03-11	*OGAPODRAFT_76806*	*CYT2*	cytochrome c1 heme lyase CYT2
TT03-12	*OGAPODRAFT_92206*	*PSA1*	mannose-1-phosphate guanylyltransferase
TT03-13	*HPODL_01049*	*GRX6*	glutathione-disulfide reductase GRX6
TT03-14	*HPODL_00042*	*RPL7A*	ribosomal 60S subunit protein L7A
TT03-15	*HPODL_04105*	*RPL42A*	ribosomal 60S subunit protein L42A
TT03-16	*OGAPODRAFT_17069*	*PTI1*	cleavage polyadenylation factor subunit PTI1
TT03-17	*HPODL_01073*	*ANB1*	translation elongation factor eIF-5A
TT03-18	*HPODL_02594*	*MMF1*	isoleucine biosynthesis protein MMF1
TT03-19	*OGAPODRAFT_102344*	*PGK1*	3-phosphoglycerate kinase
TT03-20 ^(4)^	*HPODL_02458*	*SOD2*	superoxide dismutase SOD2
TT03-21 ^(4)^	*HPODL_02693*	*PFK26*	6-phosphofructo-2-kinase
TT03-22	*HPODL_02169*	*TAF9*	transcription initiation factor TFIID subunit 9
TT03-23 ^(4)^	*HPODL_01966*	*RAD6*	E2 ubiquitin-conjugating protein RAD6
TT03-24	*HPODL_02705*	*RPL1A*	ribosomal 60S subunit protein L1A
TT03-25 ^(4)^	*HPODL_01497*	*ASC1*	guanine nucleotide-binding protein subunit beta
TT03-26	*HPODL_01957*	*MET5*	sulfite reductase (NADPH) subunit beta
TT03-27	*OGAPODRAFT_75779*	*CEP3*	centromere DNA-binding protein complex CBF3 subunit B
TT03-28	*HPODL_03364*	*RPL23B*	ribosomal 60S subunit protein L23B
TT03-29 ^(4)^	*HPODL_00942*	*RPP2B*	ribosomal protein P2B
TT03-30	*HPODL_01497*	*ASC1*	guanine nucleotide-binding protein subunit beta
TT03-31	*HPODL_02465*	*SER2*	phosphoserine phosphatase
TT03-32	*OGAPODRAFT_74529*	*STE5*	pheromone-responsive MAPK scaffold protein
TT03-33 ^(4)^	*HPODL_03495*	*ACC1*	acetyl-CoA carboxylase
TT03-34	*OGAPODRAFT_16247*	*DEG1*	pseudouridine synthase DEG1
TT03-35	*OGAPODRAFT_76195*	*STM1*	Uncharacterized protein
TT03-36	*OGAPODRAFT_17428*	*SLM1*	phosphatidylinositol 4,5-bisphosphate-binding protein
TT03-37	*OGAPODRAFT_15585*	*RPS26A*	ribosomal 40S subunit protein S26A
TT03-38	*HPODL_03366*	*SNF3*	high-affinity glucose transporter SNF3
TT03-39	*HPODL_03527*	*IDP1*	isocitrate dehydrogenase (NADP(+))
TT03-40 ^(4)^	*OGAPODRAFT_7594*	*SOM1*	mitochondrial export protein Som1
TT03-41	*HPODL_02149*	*ETR1*	trans-2-enoyl-CoA reductase
TT03-42	*HPODL_04585*	*MYO5*	myosin-5
TT03-43	*HPODL_01873*	*SBA1*	hsp90 cochaperone SBA1
TT03-44 ^(4)^	*HPODL_01380*	*PRY2*	sterol-binding protein
TT03-45	*HPODL_01021*	*RPS27B*	ribosomal 40S subunit protein S27B
TT03-46	*HPODL_02251*	n.d.	n.d.
TT03-47	*HPODL_04413*	n.d.	n.d.
TT03-48 ^(4)^	*OGAPODRAFT_16908*	n.d.	n.d.
TT03-49 ^(4)^	*OGAPODRAFT_15905*	n.d.	n.d.

^(1)^ Full data are shown in Table S1. ^(2)^ *Ogataea polymorpha* genes were identified using the BLASTN function of UniProt. Partial nucleotide sequences were used as queries. ^(3)^ *Saccharomyces cerevisiae* orthologs were identified using the BLASTP function of SGD (E-value < 1.0 × 10^−4^). The full amino acid sequences of the *O. polymorpha* gene products were used as queries. ^(4)^ Corresponding strain was reconstructed for confirmation, as shown in Figure 2.

**Table 3:** Gene enrichment analysis of the list of 59 *O. polymorpha* genes.

GO Term		False Discovery Rate (FDR)	Number of Matches
structural constituent of ribosome	GO:0003735	0.000014579	11
translation	GO:0006412	0.00028796	9
ribosome	GO:0005840	0.047143	6

## Supplementary Materials

The following are available online at https://www.mdpi.com/article/10.3390/jof7040302/s1, Table S1: Annotation of *O. polymorpha* genes obtained from colonies of heat tolerant *S. cerevisiae* expressing *O. polymorpha* cDNA.
